# Supplementary material for: Preoperative evaluation of C2 pedicle screw placement using a deep learning model: Development and validation study
Source: PLoS One. 2026 Feb 11;21(2):e0342349. doi: 10.1371/journal.pone.0342349 (PMC12893610; doi:10.1371/journal.pone.0342349)
Supplement: S1 Table — (DOCX) [file pone.0342349.s001.docx]

**Supporting information**

| Cutoff scheme | Accuracy | Sensitivity | Specificity | AUC |
| --- | --- | --- | --- | --- |
| 4.78 mm  (primary, ROC-derived) | 0.89 | 0.9 | 0.89 | 0.94 |
| 80% screw–pedicle ratio (sensitivity analysis) | 0.66 | 0.72 | 0.6 | 0.71 |

**Table S1. Sensitivity analysis of model performance under an alternative cutoff definition (All results are based on the same 3D-printed test set)**
