# Supplementary material for: Preoperative evaluation of C2 pedicle screw placement using a deep learning model: Development and validation study
Source: PLoS One. 2026 Feb 11;21(2):e0342349. doi: 10.1371/journal.pone.0342349 (PMC12893610; doi:10.1371/journal.pone.0342349)
Supplement: Attachment — Submitted filename: Response to Reviewers.docx [file pone.0342349.s003.docx]

**Manuscript ID:** PONE-D-25-56509
**Title:** Preoperative Evaluation of C2 Pedicle Screw Placement Using a Deep Learning Model: Development and Validation Study

Dear Dr. Chou and Reviewers,

We sincerely thank the Academic Editor and all reviewers for their careful evaluation and constructive comments. We have revised the manuscript thoroughly in accordance with the suggestions. All modifications are highlighted in the tracked-changes version. Below we provide a detailed, point-by-point response. Line numbers refer to the revised manuscript with track changes.

**Response to Journal Requirements**

**1. Compliance with PLOS ONE style**

We have revised the manuscript to fully comply with PLOS ONE formatting and file-naming requirements, including figure captions, references, and structure.

**2. Code sharing**

All author-generated code has been made publicly available without restrictions at: <https://github.com/Ingram66/C2-NET>

**3–5. Funding statement**

Funding information has been removed from the Acknowledgments section and retained only in the Funding Statement. The grant number has been corrected from 2024A03J074 → 2024A03J1074.

The funders had no role in study design, data collection and analysis, decision to publish, or preparation of the manuscript.

**6. Data statement**

In accordance with PLOS policy, the contact point has been changed to a non-author institutional office: The Medical Ethics Committee of Guangdong Second Provincial General Hospital (Email: [gd2hllwyh@163.com](mailto:gd2hllwyh@163.com)).

*This has been updated in the Data Statement (Lines 437–439).*

**7. Figure captions**

Separate captions for each figure have now been provided in the manuscript.

**Response to Reviewer #1**

**Comment 1**

*The 4.78-mm MPD cutoff requires stronger justification… sensitivity analyses using alternative thresholds are recommended.*

**Response**

We appreciate this important comment. We have clarified the origin and role of the 4.78-mm threshold:

**1. Derivation of 4.78 mm**
The 4.78-mm threshold was derived from our previous independent CTA-based 3D-printed study using ROC analysis and the Youden index, and was not optimized on the current dataset. In the present study, this value was used only for data labeling (MPD ≥4.78 mm as low-risk; <4.78 mm as high-risk) and not as an intraoperative decision rule. Importantly, the labels in the external test set were determined by actual screw placement outcomes on 3D-printed models, rather than by MPD measurements, thereby avoiding circular validation.

*Added in Materials and methods--Training and validation set, Lines 109-111*

*Clarified in Discussion, Lines 362-366*

**2. Sensitivity analysis**
A supplementary analysis was conducted using an alternative 4.30-mm cutoff, selected with reference to the commonly accepted 80% screw–pedicle ratio principle, rather than as a mathematically exact derivation from the 3.5-mm screw diameter. The model was retrained using this threshold, while the external test set and its labels—based on cortical breach observed in 3D-printed screw placement—remained unchanged to avoid data leakage. Under this setting, model performance decreased compared with the ROC-derived 4.78-mm threshold, which supports the robustness and clinical appropriateness of the original cutoff.

*Added in Materials and methods--Training and validation set, Lines 115-118*

*Added in Discussion, Lines 369-372*

*Results provided in Supplementary Table S1*

**Comment 2**

*Deep learning module descriptions are insufficient for reproducibility.*

**Response**

We agree that detailed methodological reporting is essential. Accordingly, we have revised the manuscript to comprehensively describe the model architecture, training strategy, and data handling procedures to facilitate reproducibility.

*Added in Materials and methods -- Deep learning model construction, Lines 176-196.*

**Comment 3**

*Risk of data leakage between left and right pedicles.*

**Response**

Dataset partitioning was performed strictly at the patient level before any preprocessing or augmentation. All slices and both left/right pedicles from the same patient were assigned exclusively to one subset (training, validation, or testing). Therefore, no image, slice, or anatomical structure from a given patient was ever present across different subsets, effectively preventing data leakage.

*Clarified in Materials and methods--Training and validation set, Lines 112-116*

**Response to Reviewer #2**

**Comment**

*The study did not consider vertebral artery anomalies and used 3D-printed models rather than real bone conditions.*

**Response**

We thank the reviewer for this important comment. We acknowledge that the present study did not incorporate vertebral artery anomalies and that the experiments were performed on 3D-printed models rather than real intraoperative bone conditions.

In this study, cases with high-riding vertebral artery or other vascular variations were intentionally excluded, and the proposed model was designed to evaluate pedicle morphology only. We have now expanded the Discussion to clarify that clinical application of the system requires integration with CTA-based vascular assessment and that prospective validation in real surgical patients will be necessary to confirm safety and generalizability.

*Added in Discussion, Lines 347-349 and 415-419*

**Response to Reviewer #3**

**Comment 1**

*Need further discussion of the 4.78-mm value and alternative cutoffs.*

**Response**

Addressed as described for Reviewer #1. A sensitivity analysis is now presented (Supplementary Table S1).

**Comment 2**

*Claim that the model outperforms junior residents is not supported (P>0.05).*

**Response**

Thank you for noting this. The statement has been corrected to reflect **no statistically significant difference** between C2-Net and junior surgeons.

*Revised in Discussion, Lines 351-355*

**Comment 3**

*External test set of 50 patients is small.*

**Response**

This limitation has been explicitly acknowledged.

*Added in Discussion, Lines 418-420*

**Comment 4**

*Need explicit discussion of data leakage.*

**Response**

Addressed as above;

*Clarified in Materials and methods--Training and validation set, Lines 112-116*

**Supplementary Material**

| Cutoff scheme | Accuracy | Sensitivity | Specificity | AUC |
| --- | --- | --- | --- | --- |
| 4.78 mm  (primary, ROC-derived) | 0.89 | 0.9 | 0.89 | 0.94 |
| 80% screw–pedicle ratio (sensitivity analysis) | 0.66 | 0.72 | 0.6 | 0.71 |

Table S1. Sensitivity analysis of model performance under an alternative cutoff definition (same 3D-printed test set)

We sincerely appreciate the reviewers’ valuable suggestions, which have substantially improved the manuscript. We hope the revised version is now suitable for publication in *PLOS ONE*.

Sincerely,
Li Zhang
